# Supplementary material for: Undefeated—Changing the phenamacril scaffold is not enough to beat resistant Fusarium
Source: PLoS One. 2020 Jun 29;15(6):e0235568. doi: 10.1371/journal.pone.0235568 (PMC7323951; doi:10.1371/journal.pone.0235568)
Supplement: S1 File — (PDF) [file pone.0235568.s001.pdf]

Supplementary materials

Undefeated – Changing the phenamacril scaffold is not enough to beat resistant Fusarium

Rasmus D. Wollenberg, Søren S. Donau, Manuel H. Taft, Zoltan Balázs, Sven Giese, Claudia Thiel, Jens L. Sørensen, Thorbjørn T. Nielsen, Henriette Giese, Dietmar J. Manstein, Reinhard Wimmer and Teis E. Sondergaard

TABLE S1. Oligonucleotide primers used in this study.

Primer ID	Sequence 5´-3´	
P1410-F	CAAACTACCTCCTCGAAAGATCACGAGTGGTGGGCCAGATC	K216R
P1411-R	GATCTGGCCCACCACTCGTGATCTTTCGAGGAGGTAGTTTG	
P1412-F	CAAACTACCTCCTCGAAGAATCACGAGTGGTGGGCCAGATC	K216E
P1413-R	GATCTGGCCCACCACTCGTGATTCTTCGAGGAGGTAGTTTG	
P1414-F	CAAACTACCTCCTCGAAAAATTACGAGTGGTGGGCCAGATC	S217L
P1415-R	GATCTGGCCCACCACTCGTAATTTTTCGAGGAGGTAGTTTG	
P1416-F	CAAACTACCTCCTCGAAAAACCACGAGTGGTGGGCCAGATC	S217P
P1417-R	GATCTGGCCCACCACTCGTGGTTTTTCGAGGAGGTAGTTTG	
P1418-F	TTCGAGAAGAACAGATTTGAACAGCTGTGCATTAATTATGTCAACG	S418R
P1419-R	CGTTGACATAATTAATGCACAGCTGTTCAAATCTGTTCTTCTCGAA	
P1420-F	CGAGAAGAACAGTTTTAAACAGCTGTGCATTAATTATGTCAACG	E420K
P1421-R	CGTTGACATAATTAATGCACAGCTGTTTAAAACTGTTCTTCTCG	
P1422-F	CGAGAAGAACAGTTTTGGACAGCTGTGCATTAATTATGTCAACG	E420G
P1423-R	CGTTGACATAATTAATGCACAGCTGTCCAAAACTGTTCTTCTCG	
P1424-F	CGAGAAGAACAGTTTTGACCAGCTGTGCATTAATTATGTCAACG	E420D
P1425-R	CGTTGACATAATTAATGCACAGCTGGTCAAAACTGTTCTTCTCG	
P1428-F	CGAAAGCAGCCTCCCTCTGGAGGTGACCGTATCCGAGCC	A577G
P1429-R	GGCTCGGATACGGTCACCTCCAGAGGGAGGCTGCTTTCG	
P1168-F	GGTCTTAAUACATAAGCAGAAACTGATCATCGT	Myo5 U1
P1185-R	GGCATTAAUAAGAATAAAAACCAAACTCGGC	MR1
P1186-F	GGACTTAAUCATGCTTTGGGGCAAGCGTA	MF2
P1187-R	GGGTTTAAUGATGGACAAAGGTCCCAAGGAGTG	MR2
P1472-F	AGTGTGTCTGGTGGAGAATC	MyoMutF
P1473-R	AGGTGTCCATTGGATCTG	MyoMutR

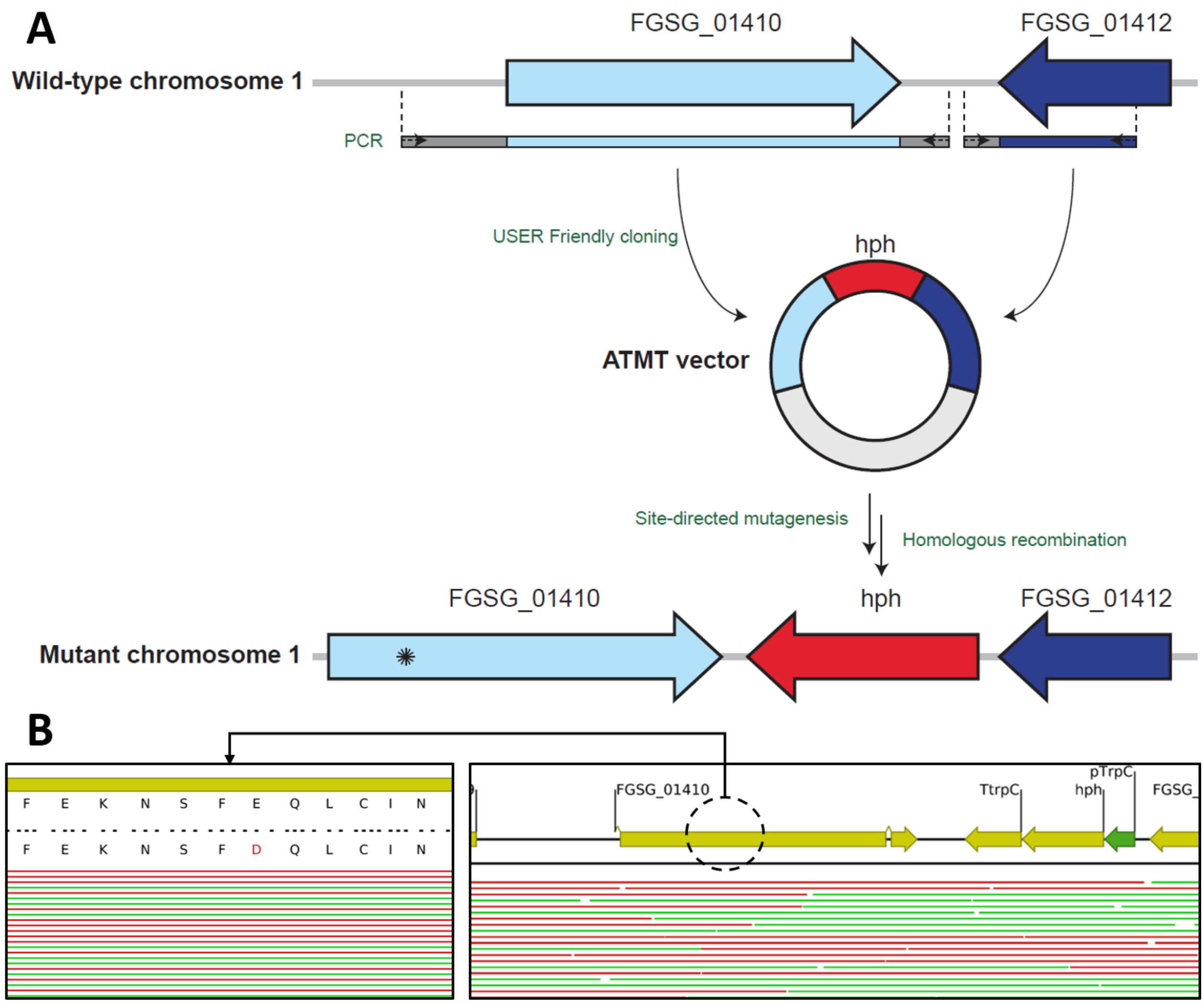

FIG S1. (**A**) Cloning strategy for introducing point mutations into the *myosin5* locus (FGSG_01410) of *F. graminearum* PH-1. ATMT: *Agrobacterium tumefaciens* mediated transformation, hph: Hygromycin-B-phosphotransferase coding sequence. (**B**) Mapping of Oxford Nanopore sequencing reads from mutant E420D to the mutant *in silico myosin-5* locus (with wild-type *myosin-5* cds). Translation of the consensus sequence highlights the E420D mutation that is present in this mutant.

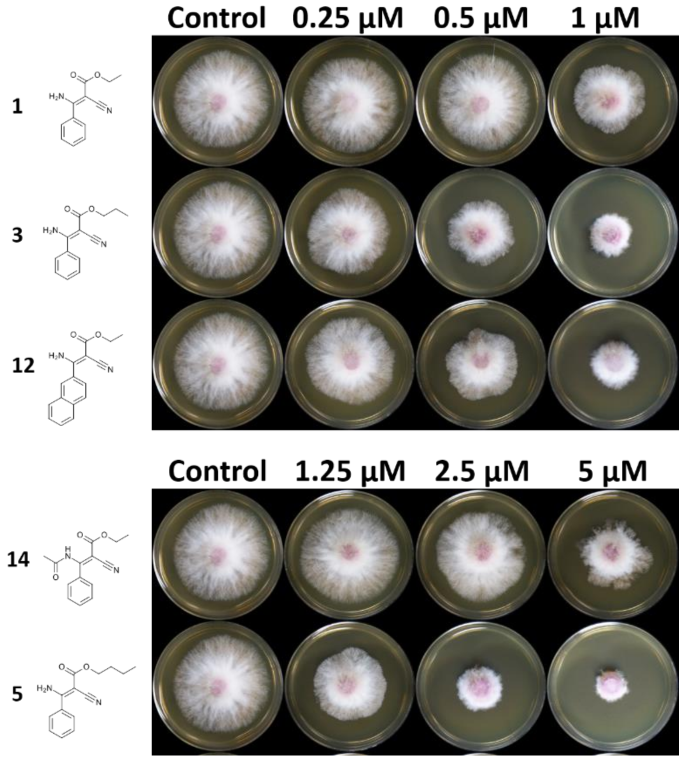

FIG S2. Amended agar-assay of compounds **1** (Phenamacril), **3**, **12**, **14** and **5** at 250 nM-5000 nM, highlighting the subtle differences in EC_{50} values for compounds with sustained growth-inhibitory activity.

SUPPLEMENTARY METHODS

Undefeated – Changing the phenamacril scaffold is not enough to beat resistant *Fusarium*

Rasmus D. Wollenberg, Søren S. Donau, Manuel H. Taft, Zoltan Balázs, Sven Giese, Claudia Thiel, Jens L. Sørensen, Thorbjørn T. Nielsen, Henriette Giese, Dietmar J. Manstein, Reinhard Wimmer and Teis E. Sondergaard

Compounds

	Structure(s)	Synthesis	Yield	Isomer	Ratio (*E*) : (*Z*)
**1**	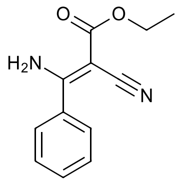	A	87.8%	(*Z*) *	- (***)
**2**	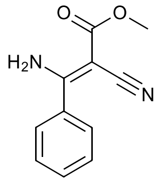	A	84.1%	(*Z*) **	- (***)
**3**	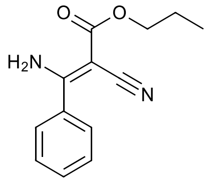	B	50.9%	(*Z*) **	- (***)
**4**	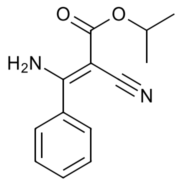	A	25.1%	(*Z*) **	- (***)
**5**	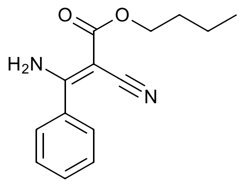	B	43.6%	(*Z*) **	- (***)
**6**	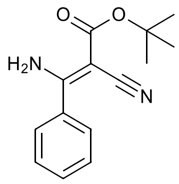	B	88.0%	(*Z*) **	- (***)

**7**	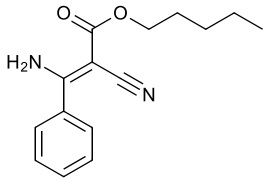	B	26.0%	(*Z*) **	- (***)
**8**	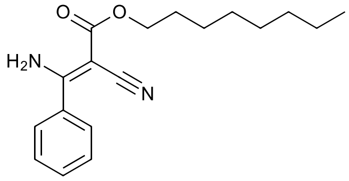	B	33.4%	(*Z*) **	- (***)
**9**	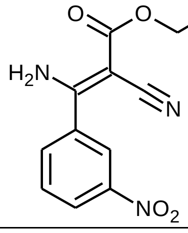	B	29.3%	(*Z*) **	- (***)
**10**	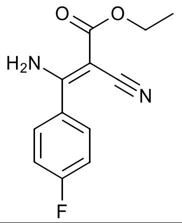	B	12.9%	(*Z*) **	- (***)
**11**	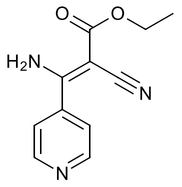	B	39.5%	(*Z*) **	- (***)
**12**	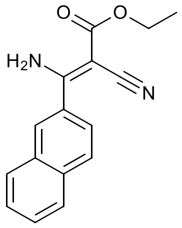	A	47.0%	(*Z*) **	- (***)
**13**	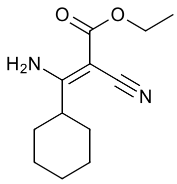	A	20.0%	(*Z*) **	- (***)
**14**	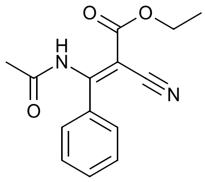	C	68.9%	(*Z*) **	- (***)

**15**	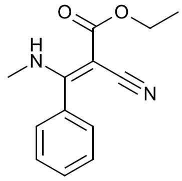	D	11.2%	(*Z*) *	- (***)
**16**	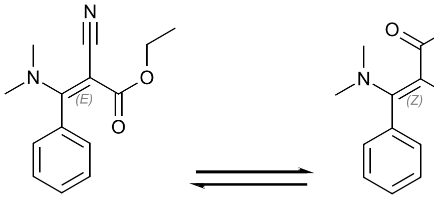	D	88.8%	(*Z*) and (*E*) * (dynamic equilibrium)(1)	≈1 : 1
**17**	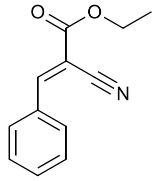	E	76.4%	(*E*) *	-
**18**	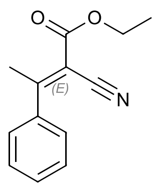 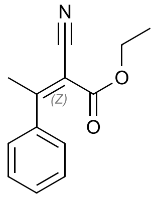 **18a** and **18b**	F	18.8%	(*E*) and (*Z*) *	31:69
**19**	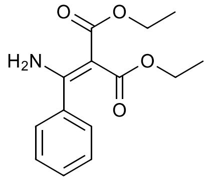	G	57.4%	-	-

* The structure and isomeric configuration of the compounds were previously described by Donau et al.(1)

** The structures and isomeric configuration of the compounds were determined by chemical shifts comparison to those of compound **1** and **15** – **19.**

*** When nothing stated, only the mentioned isomer was observed in the NMR spectrum.

IUPAC names

1 (*Z*)-Ethyl 2-cyano-3-amino-3-phenylacrylate

2 (*Z*)-Methyl 2-cyano-3-amino-3-phenylacrylate

3 (*Z*)-Propyl 2-cyano-3-amino-3-phenylacrylate

4 (*Z*)-Isopropyl 2-cyano-3-amino-3-phenylacrylate

5 (*Z*)-Butyl 2-cyano-3-amino-3-phenylacrylate

6 (*Z*)-*tert*-Butyl 2-cyano-3-amino-3-phenylacrylate

7 (*Z*)-Pentyl 2-cyano-3-amino-3-phenylacrylate

8 (*Z*)-Octyl 2-cyano-3-amino-3-phenylacrylate

9 (*Z*)-Ethyl 2-cyano-3-amino-3-(3-nitrophenyl)acrylate

10 (*Z*)-Ethyl 2-cyano-3-amino-3-(4-fluorophenyl)acrylate

11 (*Z*)-Ethyl 2-cyano-3-amino-3-(pyridin-4-yl)acrylate

12 (*Z*)-Ethyl 2-cyano-3-amino-3-(2-naphtyl)acrylate

13 (*Z*)-Ethyl 2-cyano-3-amino-3-cyclohexylacrylate

14 (*Z*)-Ethyl 2-cyano-3-acetamido-3-phenylacrylate

15 (*Z*)-Ethyl 2-cyano-3-(methylamino)-3-phenylacrylate

16 (*Z/E*)-Ethyl 2-cyano-3-(dimethylamino)-3-phenylacrylate

17 (*E*)-Ethyl 2-cyano-3-phenylacrylate

18 (*Z/E*)-Ethyl 2-cyano-3-methyl-3-phenylacrylate

19 Diethyl (2-(2-amino-2-phenyl methylidene)) malonate

Preparation of compounds

A. The syntheses of compound **1**, **2, 4, 12** and **13** were performed using an alcohol solvent and with the cyanoacetate corresponding to the desired ester moiety. For compound **1, 12** and **13** that is ethanol and ethyl cyanoacetate, for **2** the solvent was methanol and methyl cyanoacetate and for **4;** isopropanol and isopropyl cyanoacetate. For compound **2**, the lower boiling point of methanol resulted in an adjusted temperature of to 65°C. For compound **12** and **13**, the imidates were produced from nitriles by the pinner synthesis.

*General synthetic method for preparing imidates (using compound **12** as example).*

2-cyanonapthalene (2g, 19.2 mmol, 97%, Sigma Aldrich) was dissolved in dry ethanol (3 mL, anhydrous, 99.9% Vol, CCS Heathcare AB – additionally dried over molecular sieves (3Å, Sigma Aldrich)). The solution was cooled to 0 °C. Approx. 20 equivalents of dry hydrochloric acid were slowly lead through the reaction mixture over a period of approx. 5 hours, while the temperature was kept at 0 °C. The reaction was sealed and kept overnight at room temperature. Product was observed as white needle crystals and excess hydrochloric acid and solvent was removed *in vacuo*.

*General procedure (using compound **1** as example)*

Ethyl benzimidate hydrochloride (2.50 g, 12.8 mmol, 97%, Sigma Aldrich) was dissolved in dry ethanol (10 mL, 99.9% (Vol.), CCS Healthcare AB – additionally dried over 3 Å molecular sieves (Sigma Aldrich)(2)) and ethyl cyanoacetate (1.5 mL, 13.8 mmol, >98%, A-Aldrich Chemistry) was added. The solution was cooled 0°C and while stirred vigorously, freshly distillated triethyl amine (4.5 mL, 32.1 mmol, >99%, Sigma Aldrich) was slowly (9 mL h^{-1}) added. The solution was kept at 0°C for 15 minutes and then heated to 75°C under N_2 atmosphere for 15 hours.

*General workup (compound **1** and **2**)*

Water (10 mL) was added the solvents reduced *in vacuo* to a final volume of approx. 10 mL. The aqueous solution was cooled to 0°C for 1 hour and the formed precipitate was filtered and washed with 3 x 10 mL cold (0°C) water. The resulting white crystals were resolubilized in a minimum of hot acetone and recrystallized in cold (0°C) water.

*Column Chromatography (**4**, **12** and **13**)*

Automated column chromatography (Büchi Reveleris PREP Purification System) were performed for compound **4, 12** and **13** on a silica column using a hexane:EtOAc:MeOH (79:18:3) eluent.

B. Compound **3 – 11** were produced by a condensation of benzamidine with ethyl cyanoacetate in ethanol, following the procedure reported by Kenner and co.-workers.(3) For compound **3, 4, 5, 7** and **8,** ethanol was replaced by an alcohol solvent corresponding to the desired ester moiety. Compound **6** was prepared in *tert*-butanol (Sigma Aldrich) with *tert*-butyl cyanoacetate (Sigma Aldrich). For compound **9**, **10** and **11,** a benzamidine having the desired functionality in *para*-position was used. All chemicals were provided by Sigma Aldrich.

General Workup

Automated column chromatography (Büchi Reveleris PREP Purification System) was performed for compound **3** – **11** on a silica column using Dichloromethane (Sigma Aldrich) as eluent.

C. Synthesis of compound **14** (N-Acetylation).

Phenamacril, **1**, (0.25 g, 1.2 mmol) was dissolved in acetic anhydride (2 mL) and heated to 60°C and concentrated H_2SO_4 (5 µL) was added. After 30 minutes, the solution was quenched with water. Extra water (10 mL) was added and the product extracted with dichloromethane (3x10 mL). The combined organic phases were washed with saturated $NaHCO_3$ and brine and dried over $MgSO_4$. Solvent was reduced *in vacuo* and the product purified automated flash column chromatography (silica, eluent; dichloromethane).

D. Synthesis of compound **15** and **16** (N-methylation)

Phenamacril, **1**, (0.5 g, 2.3 mmol) and anhydrous potassium carbonate (3.2 g, 23 mmol) were mixed in anhydrous dimethyl formamide (10 mL). Methyl iodide (0.6 mL, 9.5 mmol) was added. After 12 hours at 20°C the reaction mixture was filtered and the liquid fraction reduced *in vacuo* at 50°C. The remains were dissolved in a minimum of heptane : ethyl acetate : methanol (79.4 : 18 : 2.6) and purified using automated column chromatography (Büchi Reveleris PREP Purification System) with an isocratic eluent (heptane (79.4%), ethyl acetate (18%), Methanol (2.6%)). The collected fractions were concentrated *in vacuo*.

E. Synthesis of compound **17**(4)

Benzaldehyde (2 g, 18.5 mmol) and ethyl cyanoacetate (2.1 g, 18.5 mmol) was mixed and piperidine (0.32 g, 3.7 mmol) added under vigorous stirring as the exothermic reaction initiates. Reacted overnight at room temperature where the content solidified and purified using automated column chromatography (silica, eluent; dichloromethane).

F. Synthesis of compound **18a and 18b**(4)

Acetophenoene (5 g , 41 mmol), ethyl cyanoacetate (4.7 g, 41 mmol), ammonium acetate (0.31 g, 4 mmol) and glacial acetic acid (0.5 g, 8 mmol) were mixed in toluene. The solution was heated to 105°C. Solvent was reduced *in vacuo*. The remaining oil dissolved in dichloromethane (50 mL) and washed with sat. $NaHCO_3$ (50 mL) and brine (3 x 50 mL). Solvents were reduced *in vacuo* and the compound purified (but not separated) using automated flash chromatography (silica, eluent; pentane : EtOAc at a gradient of; 0% - 30% EtOAc over 10 column volumes).

G. Compound **19** was prepared following the procedure reported by Scavo and Helquist.(5) Purified using automated flash chromatography (silica, eluent; dichloromethane).

NMR analysis:

NMR data were recorded by dissolving each compound in $CDCl_3$ (550 µL), with the only exception being compound **9,** which for structure validation was dissolved in DMSO-d_6 (500 µL). Reference spectra of Phenamacril, **1**, was also recorded in DMSO-d_6. For all compounds a complete set of ^{1}H-, ^{13}C-, HSQC and HMBC NMR data were measured at 298 K for samples in $CDCl_3$ and 308 K for samples in DMSO-d_6. All spectra were recorded on a BRUKER AVIII-600 MHz NMR spectrometer equipped with a 5mm CPP-TCI probe.

1. Donau, S. S., Bechmann, M., Müller, N., Nielsen, T. T., and Wimmer, R. (2017) (Z), Not (E) – An End to a Century of Confusion about the Double-Bond Stereoisomers of 3-Amino-2-cyanoacrylates. *European J. Org. Chem.* **2017**, 6408–6412
2. Williams, D. B. G., and Lawton, M. (2010) Drying of organic solvents: Quantitative evaluation of the efficiency of several desiccants. *J. Org. Chem.* **75**, 8351–8354
3. Kenner, G. W., Lythgoe, B., Todd, A. R., and Topham, A. (1943) Some reactions of amidines with derivatives of malonic acid. *J. Chem. Soc.* 10.1039/jr9430000388
4. Schwetlick, K. (2015) *Organikum: Organisch-chemisches Grundpraktikum*, 24th Ed., Wiley-VCH, Weinheim
5. Scavo, F., and Helquist, P. (1985) Preparation of α,β-dehydro-β-amino acid derivatives by tin-promoted addition of malonates to simple nitriles. *Tetrahedron Lett.* **26**, 2603–2606
