# Supplementary material for: Undefeated—Changing the phenamacril scaffold is not enough to beat resistant Fusarium
Source: PLoS One. 2020 Jun 29;15(6):e0235568. doi: 10.1371/journal.pone.0235568 (PMC7323951; doi:10.1371/journal.pone.0235568)
Supplement: Attachment — Submitted filename: Rev comments.docx [file pone.0235568.s002.docx]

We would like to thanks the reviewers for the comments.

Reviewer #1: The manuscript is very well described, with clear objectives and an appropriate methodology. The results of the analysis of the new molecules show us that structural changes do not always cause improvements in biological activity / toxicity. It is very difficult to find a chemical analog with an activity superior to the base compound, however planned and rational the synthesis process may be. Some less adjustments are needed:

- Results and discussion: lines 182/183/231/233, correct: (Error! Reference source not found.). Thanks, corrected.

- Line 197: lots of space. Corrected.

- Lines 253/256: Missed mentioning the figure number. Corrected

- The quality of the chemical structures in figures 3 and 4 is poor. We have made new figures 3 and 4 in better quality.

Reviewer #2: I found the manuscript very intriguing in its hypotheses, and I’m very supportive with researches aimed at demonstrating using indirect strategies or studies. Unfortunately, I have many conceptual concerns about the present manuscript, reported as follows:

Mat&Met: The section about the agar assay lacks of the culture incubation conditions (temperature, light/dark, approximate number of incubation days). It is a key information that must be provided. Additionally, no statistical analysis of growth-inhibition data seems to have been performed, since neither information about analysis software is described nor p values are indicated in charts or their legends, while something suddenly appears in Line 232 (“were slightly more potent than phenamacril (1) (P < 0.05).”)

We have added the following: These were inoculated with mycelia plugs (5 mm) from actively growing cultures. The inoculated plates were grown in dark as three replicates at 25°C in an INCU-Line incubator (VWR, Denmark) with natural convection. Growth was monitored until the controls reached the edge of the plates (2-5 days depending on the species).

And added a new paragraph: “**Statistical analysis**

All plate and ATPase assays were done in triplicates. Error bars denote mean ± S.D. Student's two-tailed t test was used to compare sample means, with statistical significance p > 0.05.”

The chemical part is very wanting. The characterisation of the compounds has been made only by NMR and no reaction yields are reported.

We have added all NMR spectra in supplemental materials and added the yield to the table in supplemental methods.

Results & Discussion
In this paragraph there are at least 5 references missing (Error! Reference source not found)

Corrected.

An evaluation of inhibitory effect of compounds was described. Although visual data were shown as pictures of strains cultures, no measurement was conducted to numerically estimate differences in growth rate…thus, I wonder how Authors could discuss and then correlate such differences to the biological activity of compounds (for example in Line 192: “branching of the aliphatic chain reduced the fungicidal potency by ≈100-fold”).

We agree that 100 fold is an unsuccessful term to use. It was meant that the inhibitory effect changed from 1uM to 100 uM. We have canceled the numbers.

Another consideration I would like to raise is about the “fungicide” potential of compounds: standing to a simple radial growth assay, as reported in the present work, it is not possible to distinguish a fungicidal activity from a fungistatic one. In fact, as frequently demonstrated, inhibitory effect of bioactives on fungi often rely on a strong delay of hyphae elongation, To be able to assess the difference, viability assay and biomass accumulation measurement should be done.

We completely agree and we have previous (ref. 4) been concerned about that fact (4. Wollenberg RD, Donau SS, Nielsen TT, Sorensen JL, Giese H, et al. (2016) Real-time imaging of the growth-inhibitory effect of JS399-19 on Fusarium. Pestic Biochem Physiol 134: 24-30. Based on our observation and combined with the literature, we find that Phenamacril possess both fungicidal and fungistatic effects dependent by concentration.

Minor issues:
Line 135: “w/wo” is this abbreviation allowed by the Journal? Changed
Line 182, 188, 231, 233, 241: reference error highlighted. It refers to figures because of automated track not recognized in PlosOnes system. Changed.
Line 253, 256: figure number is missing Changed
Figure 4: the relative position of the resistance-conferring mutations in the homology model is shown, but it is not clear if this docking analysis was performed by Author specifically for this work (if this is the case, methodological information in the proper section is missing) or if it is reported for a reader’s better comprehension only.

Methodological information has been added

***In silico* modeling**

The crystal structure of *D. discoideum* myosin-1E (PDB ID 1LKX) (Kollmar *et al.*, 2002) was used as template for homology modelling of the *F. graminearum* class I myosin motor domains in the pre-powerstroke state. The C chain of 1LKX with ADP-vanadate was energy-minimized and refined in YASARA Structure and WHAT IF ver. 17.8.15 (Yasara2 forcefield, 25°C, TIP3P water model, 1000 ps simulation, 40 snapshots) (Krieger and Vriend, 2014). The lowest-energy conformer was subsequently used as a template in SWISS-MODEL (Bordoli *et al.*, 2009). Following re-positioning of the ADP-vanadate, the model was refined using the above parameters.

Three refs from this section have been added to the literature list.
