# Supplementary material for: Undefeated—Changing the phenamacril scaffold is not enough to beat resistant Fusarium
Source: PLoS One. 2020 Jun 29;15(6):e0235568. doi: 10.1371/journal.pone.0235568 (PMC7323951; doi:10.1371/journal.pone.0235568)
Supplement: Attachment — Submitted filename: rev comments2.docx [file pone.0235568.s003.docx]

A revised version of this manuscript should address the following points:

1. Statistical analyses: Line 190 says n = 3, but in line 427 it says n = 6. Corrected, it was six replicates

2. Fungicidal vs. fungistatic activity: I appreicate your response to the issue raised by reviewer #2. However, I can't see that this has point has been taken into account in the revised manuscript. If you believe that the reviewer's comment can be addressed by previous results/studies, then this needs to be discussed in the main text with appropriate literature being referenced.

We have added the following to results and discussion:

It is difficult to distinguish between fungicidal and fungistatic activity working with fungicides. In the case Phenamacril, we have previous demonstrated that Phenamacril predominately works in a fungistatic way in concentrations lower than 100µM inhibiting elongation of hyphae [4].

3. Line 191: "Student's two-tailed t test was used to compare sample means, with statistical significance p > 0.05."
Presumably, this should be p <= 0.05. Corrected

4. Line 424: "Derivatives 8 and 9 were due low solubility assayed at 0, 1, 10 and 20 μM."
Please amend to: Derivatives 8 and 9 were assayed at 0, 1, 10 and 20 μM due low solubility. Changed

5. Supplementary methods, compounds table: yield should be given with only one significant decimal after the floating point or as integer values. Corrected

6. Throughout (manuscript and supplemental information):
a. Please use consistent upper or, preferrably, lower case 'p' to denote the p-value. Corrected
b. Consistently add space between the number and '°C' when giving temperatures(e.g. '0 °C' instead of '0°C'). Corrected
c. check spelling, in particular the name of chemicals (e.g. dichloromethane instead of Dichloromethane). Corrected
d. Variables should be set in italics, e.g. <i>t</i>-test, <i>p</i>-value, etc. Corrected
